# Transjugular Approach to Closure of Patent Foramen Ovale Under the Guidance of Fluoroscopy and Transthoracic Echocardiography: A Case Report

**DOI:** 10.3389/fcvm.2022.905614

**Published:** 2022-05-20

**Authors:** Lu He, Jian-ying Xue, Ya-juan Du, Xue-gang Xie, Xing-ye Wang, Yu-shun Zhang

**Affiliations:** Department of Structural Heart Disease, The First Affiliated Hospital of Xi'an Jiaotong University, Xi'an, China

**Keywords:** patent foramen ovale, inferior vena cava thrombosis, closure, transjugular, atrial septal aneurysm

## Abstract

**Background:**

We describe a rare case of patent foramen ovale (PFO) associated stroke in a patient with pulmonary embolism, inferior vena cava thrombosis and undergoing filter implantation who successfully underwent PFO closure using the right internal jugular venous approach.

**Case Summary:**

This is a rare case of a 42-year-old patient who presented with stroke and pulmonary embolism and was diagnosed with a PFO, inferior vena cava thrombosis and underwent filter implantation. The patient suffered from stroke and pulmonary embolism successively; that is, embolic events occurred in both the arterial and venous systems. Transesophageal echocardiography (TEE) showed a PFO with an atrial septal aneurysm (ASA), which we considered a “pathological” PFO. Due to the obstructive nature of the inferior vena cava approach, we successfully performed PFO closure via the right internal jugular venous approach under the guidance of X-ray and transthoracic echocardiography (TTE).

**Discussion:**

The right jugular venous approach provides a simple technical solution for patients who require PFO closure when femoral venous access is unavailable, which can be performed under X-ray and TTE guidance.

## Introduction

Transcatheter closure of a patent foramen ovale (PFO) is usually performed using a femoral venous approach. This procedure becomes more difficult when femoral vein access is not feasible due to congenital or acquired reasons. There are only a few reports of PFO closure via the jugular venous approach in this special case, and these procedures were mostly performed under general anesthesia and under the guidance of transesophageal echocardiography (TEE) or intracardiac echocardiography (ICE) (1–3). We report a case of PFO associated stroke in a patient with atrial septal aneurysm (ASA), pulmonary embolism, inferior vena cava thrombosis and filter implantation, under the guidance of local anesthesia, X-ray and transthoracic echocardiography (TTE), the patient was successfully underwent PFO closure using the right internal jugular venous approach.

## Case Presentation

**Figure 1:**
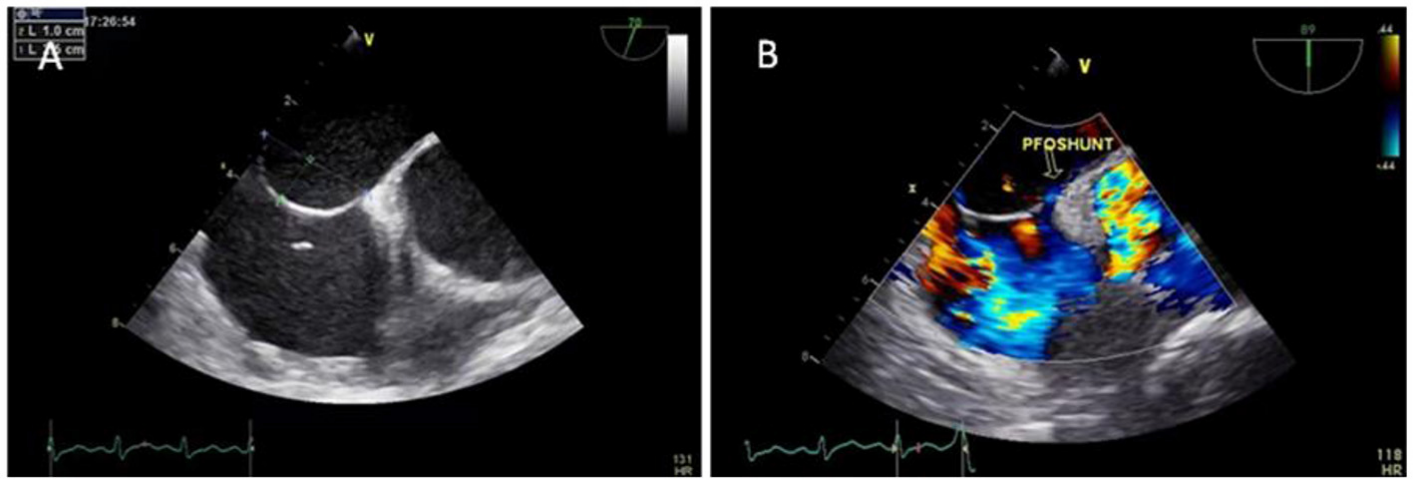
TEE showed a PFO with an ASA **(A)** and a small amount of left-to-right shunt through the fossa ovale **(B)**.

A 42-year-old male with sudden left upper extremity dysfunction was diagnosed with cerebral infarction by MRI and cerebral angiography. Intracerebral hemorrhage occurred after intravenous thrombolysis. One month later, a CT scan of the pulmonary artery was performed because the patient complained of left chest pain, shortness of breath and lower extremity edema, which indicated extensive thrombosis of the bilateral pulmonary arteries. Ultrasound of bilateral lower extremity veins showed left femoral vein, superficial femoral vein, and popliteal vein thrombosis. To prevent massive pulmonary embolism, anticoagulation therapy (rivaroxaban 20 mg/d) and inferior vena cava filter implantation were performed. Antithrombin III, protein C, protein S, tumor markers, antiphospholipid antibodies, autoantibodies were all negative. Because the patient has successively experienced embolism of the arterial system and venous system without risk factors, such as hypertension, diabetes, smoking and so on, paradoxical embolism cannot be excluded. TEE examination revealed a large PFO with an ASA (Figure 1). After multidisciplinary diagnosis, transcatheter PFO closure is recommended.

**Figure 2:**
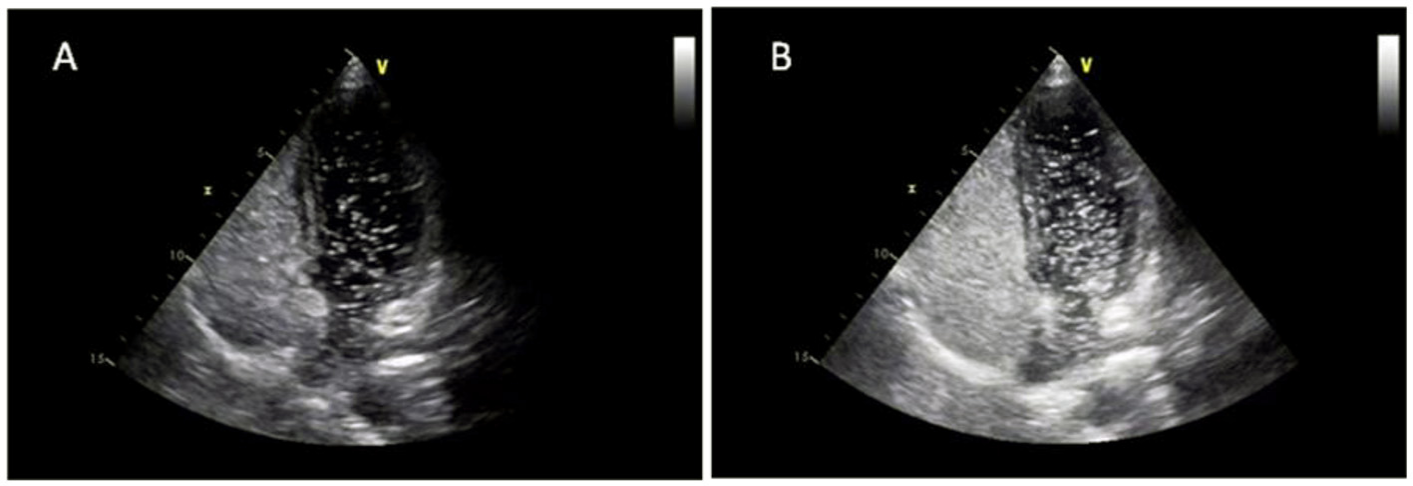
cTTE showed substantial RLS at rest **(A)** and Valsalva maneuver **(B)**.

**Figure 3:**
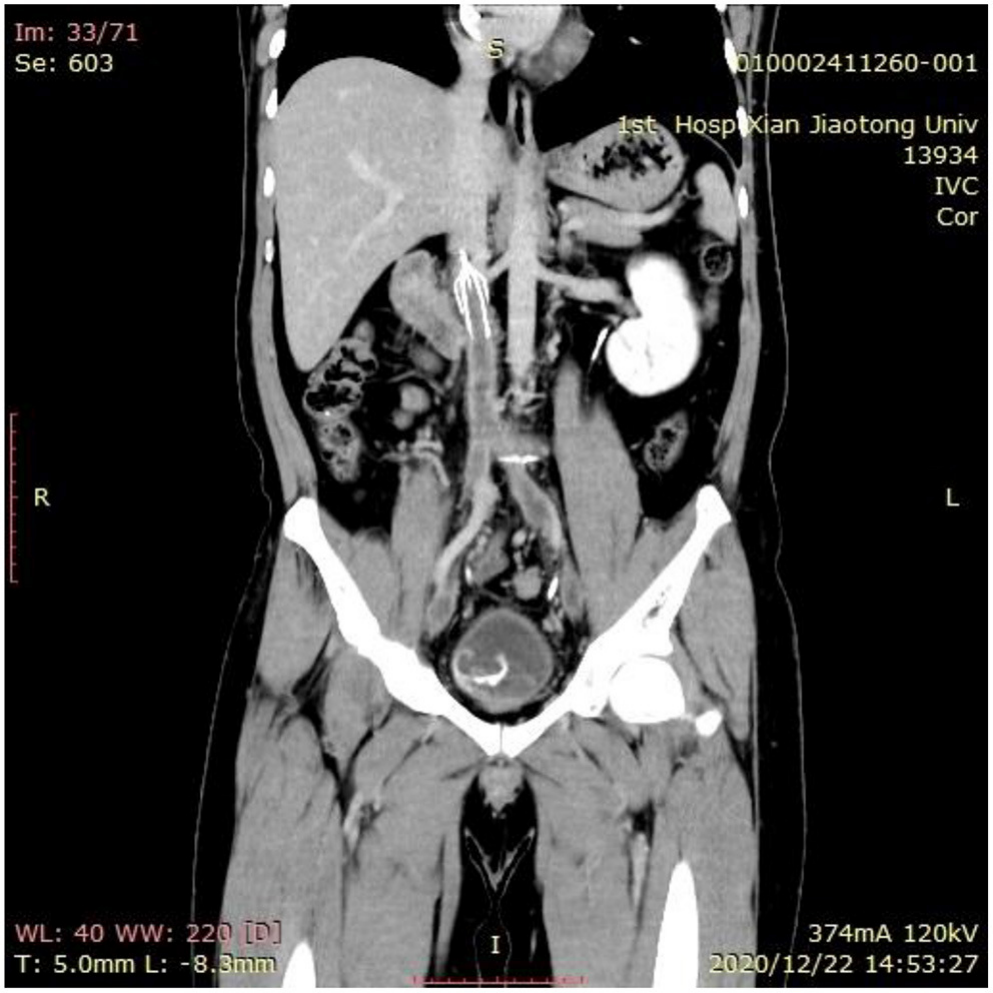
The CT examination of the lower extremity veins showed implantation of the inferior vena cava filter, inferior vena cava and bilateral external iliac vein thrombosis, and bilateral internal iliac vein thrombosis.

Preoperative TTE examination showed a PFO with an ASA, and contrast transthoracic echocardiography (cTTE) showed substantial right-to-left shunting (RLS) at rest and Valsalva maneuver (Figure 2). The CT examination of the lower extremity veins showed implantation of the inferior vena cava filter, inferior vena cava and bilateral external iliac vein thrombosis, and bilateral internal iliac vein thrombosis (Figure 3).

**Figure 4:**
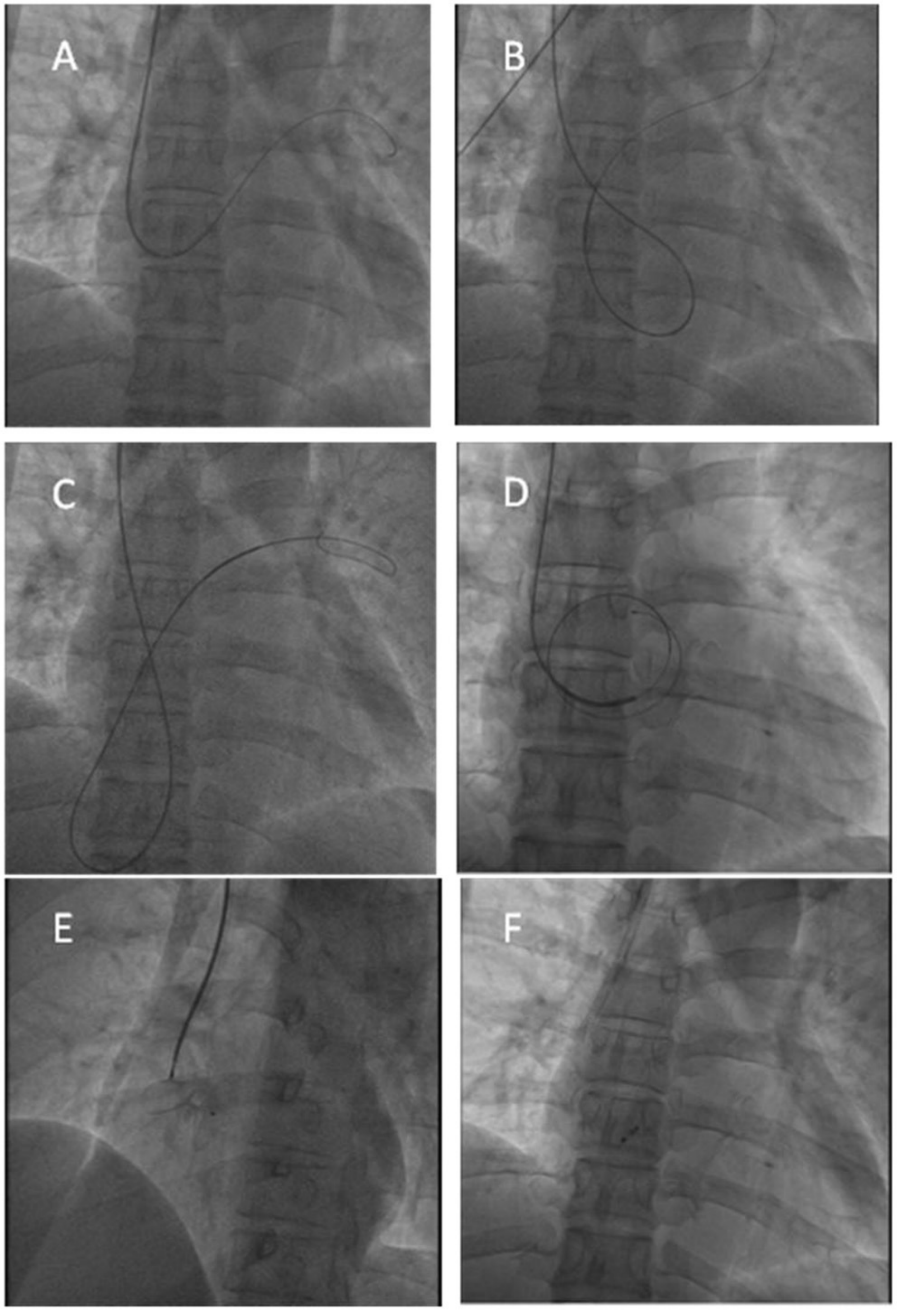
Transcatheter PFO closure procedure. **(A,B)** The guidwire reached the left superior pulmonary vein through the PFO; **(C,D)** The ordinary guidewire could not provide sufficient support, and a 180 cm 0.025” stainless steel guidewire with coiled floppy tip was exchanged to provide sufficient support; **(E)** X-ray showed good position before the occluder was released; **(F)** X-ray showed good position after the occluder was released.

Due to the conventional approach of bilateral external iliac veins, internal iliac veins and inferior vena cava all have thrombi, the superior vena cava and jugular venous systems appeared normal; therefore, a right jugular venous approach was planned. A 6 Fr sheath was placed in the right jugular vein using the Seldinger technique. Initial attempts to probe the PFO utilized multiple catheters (6Fr TERUMO Radial TIG catheter, Medtronic LAUNCHER 6Fr AL catheter, Cordis 6Fr JR4 catheter), and probing with a straight 0.035 inch guidewire was unsuccessful. A Medtronic LAUNCHER 6Fr EBU 3.5 mm guiding catheter was then advanced to the right atrium, initially positioned below the atrial septum at the level of the tricuspid valve, and then withdrawn superiorly, allowing it to engage and pass through the PFO into the left atrium and left superior pulmonary vein (Figures 4A,B). Due to insufficient support, when the guiding catheter was passed through the PFO, the guiding catheter and guidewire were slid into the inferior vena cava simultaneously (Figure 4C). Under the guidance of the guidewire, the EBU3.5 mm guiding catheter was sent to the fossa ovalis again, and a 180 cm 0.025” stainless steel guidewire with coiled floppy tip was exchanged. The guiding catheter passed through the PFO smoothly and the stainless steel guidewire was looped into the left atrium (Figure 4D). The position of the guidewire was confirmed by TTE, and there was no pericardial effusion. Withdrawing the guiding catheter, as per the classic femoral venous approach, a 9Fr Abbott TorqVue 180° delivery sheath was advanced over the wire into the left atrium, and an Amplatzer 30 mm PFO occluder (St. Jude Medical, Golden Valley, MN) was successfully deployed with good apposition as assessed by fluoroscopy and TTE (Figures 4E,F). Following device deployment, there was no evidence of residual shunt on TTE Doppler, which also showed a negative bubble study result. Recovery was uncomplicated, and the patient was discharged the next day.

## Discussion

Over the past 5 years, multiple randomized controlled trials (RCTs) have established robust evidence for preventing cryptogenic stroke (CS) by percutaneous closure of PFO (4–7). Therefore, the Amplatzer PFO occluder is indicated for percutaneous closure of a PFO to reduce the risk of recurrent ischemic stroke in patients who are predominantly between the ages of 18 and 60 years, who have had CS due to a presumed paradoxical embolism, as determined by a neurologist and cardiologist following an evaluation to exclude known causes of ischemic stroke.

The choice of approach for transcatheter PFO closure usually depends on the anatomical relationship between the vena cava and the atrial septum. Studies have shown that the angle between the inferior vena cava and the PFO tunnel is usually ~45°, which is very suitable for guiding the guidewire through the PFO to reach the left atrium, thus making it easier for the subsequent delivery sheath system to reach the left atrium, and thereby shortening the procedure time (2). Therefore, the femoral venous approach is often used as a method to easily and reliably pass across the PFO into the left atrium and, subsequently, deploy the device.

When the femoral venous approach is not possible due to inferior vena cava interruption for congenital or acquired reasons, transcatheter PFO closure by conventional methods is difficult to perform. Moreover, the number of PFO closures increases each year. In addition to the femoral venous approach, alternative approaches for PFO closure include the jugular, hepatic, and subclavian venous approaches. PFO closure through these approaches usually needs to be performed under the guidance of general anesthesia and TEE, and there are also individual reports that it is performed under the guidance of ICE (1–3).

If this patient chose the femoral venous approach, transfemoral-iliac vein intubation and balloon dilation were required first. Due to the increased risk of thrombus in the inferior vena cava along with a high-risk PFO, the risk of paradoxical embolism during the procedure is high, so the femoral venous approach is obviously not feasible. Transhepatic access has been reported in the interventional treatment of atrial septal defect (ASD) and PFO because it does not require special catheter technology (8–10). However, it requires a gelatin sponge or coils to stop bleeding through the ducts that pass through the liver, and there have been individual cases of postoperative abdominal discomfort due to peritoneal irritation caused by bleeding (11). At present, the subclavian venous approach, which has the potential to increase the risk of hemorrhage due to the large-sized sheath placed through the junction of the clavicle and the first rib, is basically not used. For these reasons, we plan to use the internal jugular vein as the preferred access point and the hepatic venous approach as an alternative.

It is technically challenging to approach the fossa ovale after entering the right atrium from the internal jugular vein because the tip of the sheath needs to have a curvature close to 90° or more to be close to the fossa ovale. Existing case reports show that various curved guiding catheters can be tried, the tip of the catheter can be shaped with cold saline, or the flexible sheath can be selected to increase the maneuverability of the catheter with the guidewire to pass through the PFO (1, 2, 12). In addition, the stiffness of the catheter tip also affects the procedure process. If the catheter tip is too soft, even if the guidewire passes through the PFO, the stiffened wire cannot be exchanged, and the delivery sheath cannot pass through the atrial septum due to a lack of strong support. If the tip is too rigid, there is a risk of injuring the left atrial wall when the catheter is passed through the atrial septum into the left atrium.

We describe a PFO associated stroke in a patient with an ASA, pulmonary embolism, inferior vena cava thrombosis and filter implantation who successfully underwent PFO closure by the guidance of X-ray and TTE using the right internal jugular venous approach for the first time. In this patient, we tried different curved catheters and failed to pass through the PFO. Then, we chose the EBU guiding catheter, and with the help of the guidewire, we reached the flaccid atrial septum and entered the left atrium through the PFO. At the same time, to solve the problem of insufficient support, we used a 180 cm 0.025” stainless steel guidewire with coiled floppy tip to pass through the interatrial septum to reach the left atrium and then successfully released the PFO occluder under the guidance of X-ray and TTE.

## Conclusion

The right jugular venous approach provides a simple technical solution in patients requiring PFO closure when femoral venous access is not available. The classic jugular venous approach to PFO closure has always been considered difficult, but we describe the use of the EBU guiding catheter and a 180 cm 0.025” stainless steel guidewire with coiled floppy tip, which enables relatively simple passage across the PFO and provides sufficient support. This technique can be performed safely under the guidance of X-ray and TTE and provides an additional option for patients who require percutaneous PFO closure without femoral or hepatic venous access.

## Data Availability Statement

The original contributions presented in the study are included in the article/supplementary material, further inquiries can be directed to the corresponding author/s.

## Author Contributions

All authors contributed to this patient care, diagnosis and treatment, to the writing of this article, and approved the submitted version.

## Conflict of Interest

The authors declare that the research was conducted in the absence of any commercial or financial relationships that could be construed as a potential conflict of interest.

## Publisher's Note

All claims expressed in this article are solely those of the authors and do not necessarily represent those of their affiliated organizations, or those of the publisher, the editors and the reviewers. Any product that may be evaluated in this article, or claim that may be made by its manufacturer, is not guaranteed or endorsed by the publisher.
